# Neural Mechanisms of Positive Mood Induced Modulation of Reality Monitoring

**DOI:** 10.3389/fnhum.2016.00581

**Published:** 2016-11-15

**Authors:** Karuna Subramaniam, Jeevit Gill, Patrick Slattery, Aditi Shastri, Daniel H. Mathalon, Srikantan Nagarajan, Sophia Vinogradov

**Affiliations:** ^1^Department of Psychiatry, University of California San FranciscoSan Francisco, CA, USA; ^2^Department of Radiology and Biomedical Imaging, University of California San FranciscoSan Francisco, CA, USA

**Keywords:** fMRI, medial prefrontal cortex, positive mood induction, reality monitoring, source memory

## Abstract

This study investigates the neural mechanisms of mood induced modulation of cognition, specifically, on reality monitoring abilities. Reality monitoring is the ability to accurately distinguish the source of self-generated information from externally-presented contextual information. When participants were in a positive mood, compared to a neutral mood, they significantly improved their source memory identification abilities, particularly for self-generated information. However, being in a negative mood had no effect on reality monitoring abilities. Additionally, when participants were in a positive mood state, they showed activation in several regions that predisposed them to perform better at reality monitoring. Specifically, positive mood induced activity within the medial prefrontal cortex (mPFC) and posterior cingulate cortex (PCC) was associated with improvements in subsequent identification of self-generated information, and positive mood induced activation within the striatum (putamen) facilitated better identification of externally-presented information. These findings indicate that regions within mPFC, PCC and striatum are sensitive to positive mood-cognition enhancing effects that enable participants to be better prepared for subsequent reality monitoring decision-making.

## Introduction

Being in a positive mood state results in broader attention, broader thought-action repertoires, greater cognitive flexibility and heightened creativity (Isen et al., 1985, 1987, 1991; Estrada et al., 1994; Isen, 1999; Fredrickson, 2004; Baumann and Kuhl, 2005). Therefore, delineating the neural system mechanisms by which a positive mood influences cognition (e.g., Subramaniam et al., 2009) is critical for developing innovative treatment approaches for individuals with psychiatric illnesses characterized by deficits in positive mood and cognition, such as people suffering from depression, anxiety disorders and schizophrenia.

In this study, we examine the behavioral and neural impact of mood on reality monitoring abilities. Reality-monitoring is a special type of self-referential source memory. Reality monitoring is defined as the ability to distinguish the contextual source of internal experiences (self-generated information) from outside reality (external information; Bentall et al., 1991; Johnson et al., 1993; Morrison and Haddock, 1997; Vinogradov et al., 1997, 2008; Keefe et al., 1999; Subramaniam et al., 2012b). Several prior neuroimaging studies have shown that the medial prefrontal cortex/anterior cingulate cortex (mPFC/ACC) is a key region that supports reality monitoring and self-referential processing (Frith and Frith, 1999; Cabeza et al., 2004; Northoff et al., 2006; Vinogradov et al., 2006, 2008; Gilbert et al., 2007; Subramaniam et al., 2012b). One strategy that participants may use to identify the source of externally-presented information is to reject that the information is self-generated, and must consequently be derived from an external source. Therefore, identifying the source of external experiences can be viewed as the other side of the same coin. In fact, prior studies and ours have shown that the mPFC supports separate representations of “self” and “other”; but has been shown to be particularly implicated in tagging information as being relevant to the “self” (Ochsner et al., 2004, 2005; Amodio and Frith, 2006; Vinogradov et al., 2006, 2008). For example, Cabeza et al. (2004) also found that mPFC activation was greater when subjects viewed photographs of a building that they themselves had taken (the autobiographical “self” condition) vs. when they viewed photographs of the same building taken by another person (the “other” condition).

Neural circuitry associated with mPFC/ACC also appears to be impacted by positive mood. In our previous fMRI study of insight-based problem-solving (Subramaniam et al., 2009), we showed that healthy participants who self-rated as higher in positive mood revealed greater activity within both dorsal and ventral regions of the mPFC and within the posterior cingulate cortex (PCC) during an anticipatory preparation period prior to problem onset, when compared to participants who self-rated lower in a positive mood. Furthermore, increased mPFC activation prior to problem onset correlated with participants’ overall problem-solving ability and with their positive mood levels. These data suggest that participants in a positive mood generated a larger mPFC preparatory signal, which in turn contributed to better problem-solving performance. However, we cannot be certain as to whether a positive mood was the primary mechanism that specifically facilitated subsequent task performance, or whether some other cognitive/physiologic characteristic that may have co-varied with positive mood (such as broader attention/working memory capacity) may have played such a causal role. Therefore, carefully designed mood induction (MI) studies in which positive and neutral mood states are both induced in each participant, are necessary to allow for causal inferences to be drawn about whether, and where in the brain, a positive mood can facilitate cognition, relative to a neutral mood.

Negative mood states have been associated with increased activity within the subgenual mPFC/ACC and amygdala (Mayberg et al., 1999; Ochsner et al., 2002). We, therefore, predicted that when participants were in a negative mood as compared to a neutral mood, they would activate mPFC and parahippcampal/amygdala cortices during the autobiographical recall of negative events. However, less is known about the neural mechanisms of how a negative mood impacts cognition; in particular, the interaction between mood induced activity in mPFC and its role in reality monitoring has never been investigated to date. However, previous research has shown that in contrast to certain cognitive-enhancing effects of a positive mood (in terms of broadening attention, memory and cognitive control), behaviorally, negative mood states such as anxiety and depression have been associated with deficits in attentional and cognitive control mechanisms (Mayberg et al., 1999; Bishop et al., 2004), often inducing a narrow scope of attention (Easterbrook, 1959). Reality-monitoring is a multifaceted process which requires components of attention, memory and cognitive control; consequently, when participants were in a negative mood state, we expected to find somewhat opposite effects (or null effects) of negative mood states on reality-monitoring, as compared to a positive mood.

In the present study we induced positive, neutral and negative mood states in each participant in order to investigate the underlying neural processing during each type of MI and how neural activity associated with different induced mood states could modulate subsequent reality monitoring performance. In general, we expected a positive mood state to modulate a network of regions, including prefrontal cortices, parahippocampal cortices and basal ganglia, consistent with previous research which has shown recruitment of these regions during positive mood states, and during overall episodic source memory retrieval (Elward et al., 2015). However, it must be noted that while many regions are important for episodic memory retrieval processes in general (including parahippocampal cortices), here we examine neural activity specifically related to reality monitoring self-generated recognition processes. Therefore, we expected that regions activated during source-memory retrieval processes common to both self-generated and externally-presented retrieval, will not be observed in our tightly-constrained self-generated vs. externally-presented recall contrast; rather we expected regions (such as the mPFC), to be recruited during specific reality-monitoring self-generated processes, consistent with Mitchell and Johnson’s (2009) review.

In light of these previous findings, we hypothesized that: (1) during positive MI as compared to neutral MI, participants will show better reality monitoring performance; (2) during positive MI when compared to neutral MI, participants will show activation in both mPFC and PCC; and (3) the enhanced mPFC preparatory signal observed during the positive vs. neutral MI will be associated with better subsequent reality monitoring self-generated identification.

## Materials and Methods

### Participants

Thirty-five healthy participants (24M, 11F) were recruited via advertisement. Mean age was 42 years, and mean education was 16 years. Inclusion criteria were: no Axis I or Axis II psychiatric disorder (SCID—Nonpatient edition), no substance dependence or current substance abuse, good general physical health, age between 18 and 60 years and English as a first language. This study was carried out in accordance with the recommendations of the NARSAD Young Investigator Selection Committee, and the University of California San Francisco (UCSF) and Northern California Institute for Research and Education (NCIRE) Committees on Human Research (CHR). All participants gave written informed consent. We conducted two experiments to induce positive, neutral and negative mood states in each participant in order to test how different mood states would impact reality monitoring abilities. We first conducted a behavioral-only MI reality monitoring task which was completed by 15 participants outside the scanner (to ensure successful MI), and then we used fMRI to map brain activation patterns while another 20 participants completed the MI reality monitoring experiment in the MRI scanner.

### Behavioral Study of the Interactions Between Mood Induction and Reality Monitoring

We used validated pictures with positive, neutral and negative valence from the International Affective Pictures System (IAPS) to induce positive, neutral and negative mood states (Lang et al., 1999). Each participant viewed 138 pictures (consisting of three blocks of 46 positive, 46 neutral and 46 negative pictures), briefly presented for 4 s, and was asked to imagine being a part of that particular picture scene. To assess whether the target mood state was induced, immediately after each picture each participant rated the intensity of their mood on three different categories of positive, negative and arousal scales, with each scale ranging from 0 to 8. For each category, the 0-end was labeled “I do not feel at all‥.” and the 8-end was labeled “I feel extremely…” Participants then completed the reality monitoring task. The reality monitoring task consisted of a word-encoding phase and a reality monitoring identification phase. In the word-encoding phase, participants were visually presented with a list of semantically constrained sentences with the structure “noun-verb-noun.” The final noun was either presented by the experimenter (e.g., *The sailor sailed the* *sea*), or left blank for subjects to write down and generate themselves and then recorded by the research assistant (e.g., *The* rabbit *ate the*___). During the reality monitoring identification phase, subjects were visually presented with noun pairs from the sentence list (e.g., *rabbit-carrot*) and had to indicate whether the second word was previously self-generated (“I made it up”) or externally-presented (“You showed it to me”; Vinogradov et al., 2008; Subramaniam et al., 2012b).

### fMRI Study of the Interaction Between Mood Induction and Reality Monitoring

For the fMRI portion of the task, we personalized the MI technique for each participant via autobiographical recall of each participant’s subjective past positive, neutral and negative experiences as this method was found to be a stronger MI technique. Specifically, we attained higher target positive and negative mood ratings when the MI was personalized for each participant (i.e., through autobiographical recall) when compared with non-personalized pictures from the IAPS (Lang et al., 1999; see Supplementary Table S1). The MI portion of the experiment has two components, one consisting of a mood-word generation phase performed outside the scanner prior to scanning, and a mood experience-recall phase performed during scanning. The instructions during the mood-word generation phase were: “I would like you to try and generate 30 positive words, 30 neutral words and 30 negative words that remind you of your past experiences. The words can be names of people, places and need not have to make sense to anyone else so long as it reminds you of your past experience. Neutral words consist of words that have little or no emotional meaning to your life. For example, names of objects are usually thought of as neutral (i.e., wall, paper, table etc). Then, for each word, I’d like you to remember that the experience associated with that word, and rate how positive, negative and how aroused you feel on a scale from 0 to 8 (i.e., 0 = “I do not feel at all‥.” to 8 = “I feel extremely…”). The arousal scale can also be thought of as an excitement/anxiety index that makes your heart rate activated.”

**Figure 1:**
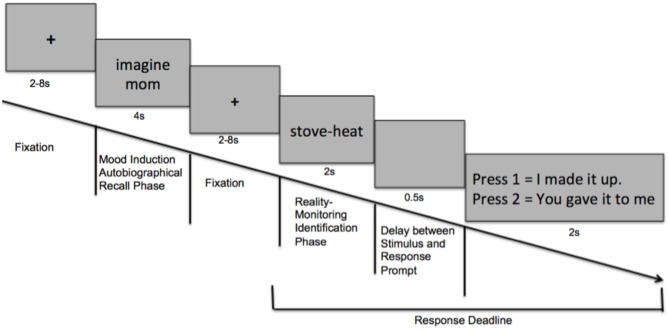
**Task design: Schematic of events within one trial of the experimental paradigm**.

During scanning, participants were shown the mood word (either positive, neutral or negative) for 4 s, and were asked to imagine the experience associated with each word in order to induce the target mood state. Using the same task as the behavioral reality-monitoring experiment, the fMRI reality monitoring task consisted of a word-encoding phase performed outside the scanner prior to scanning, and a reality monitoring identification phase performed during scanning (see Figure 1). Each fMRI run consisted of 30 trials with 30 mood-words of the same condition (i.e., 30 positive mood words, for example); 15 self-generated word pairs and 15 externally-presented word pairs randomly-presented, with each run lasting for 9 min 24 s. Participants completed a total of six runs: two positive mood conditions, two neutral mood conditions and two negative mood conditions. Order of the runs were counterbalanced so that alternating half of the participants began with the positive mood condition and ended with the negative mood condition, while the other half of participants began with negative mood condition and ended with the positive mood condition. The order sequence of the runs for one participant could thus be: positive → neutral → negative → positive → neutral → negative.

### Behavioral Statistical Analyses

The principal objective of this article is to investigate the impact of mood on source memory accuracy. Both our behavioral and fMRI MI-reality monitoring tasks showed the same pattern of efficient MI in which both positive and negative MI were successful at increasing positive and negative mood states respectively, in relation to the neutral mood condition. Therefore, to clearly demonstrate overall mood effects on source memory, we combined the behavioral analyses across the two tasks in the main article to increase the power in finding source memory interactions. (See Supplementary Table S1 and Supplementary Figure S1 for mood effects on source memory accuracy for each separate task).

Overall accuracy was summated across both correctly-identified self-generated and externally-presented information for each mood condition, and then computed as a percentage of correct responses out of the total number of source-memory items within that mood condition, averaged across the behavioral and fMRI tasks. We conducted repeated-measures analysis of variance (ANOVAs): (i) to confirm that the target mood state was successfully induced; (ii) to examine mood effects on overall accuracy; and (iii) to examine mood effects on reality monitoring self-external item identification during the positive and negative MI, relative to the neutral MI.

### Mood Induction Reality Monitoring Task: fMRI Acquisition

Visual stimuli were presented with E-Prime and back-projected onto an LCD projector. Participants viewed the screen using a mirror attached to the head coil and made finger-press responses on a fiber-optic response pad. fMRI was acquired on a 3 Tesla Tim Trio Siemens scanner and 12 channel head coil, using a Echo-planar sequence (TR = 2.4 s, 35 slices, 306 volumes, TE = 30 ms, 2 mm × 2 mm in-plane resolution, slice thickness = 3 mm, interleaved slice acquisition, FOV = 230 mm; matrix = 64 × 64).

### Mood Induction Reality Monitoring Task: fMRI Statistical Analyses

Image analysis was performed using SPM8 software^1^. Images were realigned to correct for motion artifacts using a six-parameter affine transformation, normalized to a standard stereotaxic space (Montreal Neurological Institute Template) using a 12 parameter affine/non-linear transformation, and spatially smoothed with a 8 mm Full-width half-maximum (FWHM) Gaussian kernel. The spatial resolution after normalization was 2 mm × 2 mm × 2 mm. Data were submitted to a General linear model (GLM) analysis. For each participant (i.e., first-level analysis), we fit a reference canonical hemodynamic response function (hrf) to the duration of each event within the trial (e.g., mood-word presentation, self-generated word-pair presentation (correct trials) and externally-presented word-pair presentation (correct trials)). Thus, altogether, nine event types of interest were modeled: positive mood word, neutral mood word, negative mood word, correctly identified self-generated and externally-presented item-identification in the positive mood condition, correctly identified self-generated and externally-presented item-identification in the neutral mood condition, and correctly identified self-generated and externally-presented item-identification in the negative mood condition.

Our fMRI task was designed such that variable fixation delays were used to jitter the events and optimize deconvolution of the fMRI signal from successive events, and were used as our implicit baseline (Figure 1). Further, our GLM analysis allowed us to extract signal to each trial-type, and to factor out signal due to temporally adjacent events to ensure that signal could be isolated to the event of interest. For example, when extracting signal related to MI events, we included in the analysis: the reality monitoring word-pair presentation and response presses to factor out signal tied to reality monitoring processing/outcome rather than to the MI event. We had a wait time between each run of about 1 min to allow each participant enough time to come out of the previous mood state and to start preparing for the next MI run, in order to allow complete deconvolution of the BOLD signal to baseline**.** We used the default high-pass filter cutoff in SPM8 of 128 s to account for the temporal scanner drift. Alternating participants received the positive MI first and ended with the negative mood condition, while the other half of participants began with negative mood condition and ended with the positive mood condition in order to further factor out any mood-related signal associated with scanner drift or due to participant fatigue.

Second level analyses were based on a random-effects model using a significance threshold of *p* < 0.001, uncorrected. We first conducted a one-way 3-level repeated measures ANOVA with MI condition (positive mood, neutral mood, negative mood) as the within-subject factor to test for main effects of each mood state using a significance threshold of *p* < 0.001, with a FWE cluster corrected extent at *p* < 0.05. Cluster extent based thresholding corrections minimize false positives (Type 1 errors) based on the assumption that meaningful activation is spatially clustered and is, therefore, highly sensitive, accounting for the fact that individual voxel activations are not independent of neighboring voxels (Friston et al., 2000; Woo et al., 2014; arXiv:1606.08199^2^ [stat.AP]). Next, we conducted follow-up whole-brain ANOVAs in order to examine the discrete specific neural effects of positive and negative mood states in relation to the neutral mood state (i.e., positive mood word vs. neutral mood word, and negative mood word vs. neutral mood word).

To investigate the impact of mood on reality monitoring self vs. external identification, we conducted conjunction analyses to find interactions between recruitment of brain regions during MI and reality monitoring, to confirm whether mPFC and PCC regions were specifically activated during positive mood states *and* during self-generated identification, as consistent with our predictions. As per standard SPM procedure, we conducted one sample *t*-tests in which we first masked the whole-brain positive mood effect during the MI autobiographical phase (positive vs. neutral mood state, thresholded at *p* < 0.001) as well as the whole-brain self-generated identification effect during the reality-monitoring identification phase (self-generated vs. externally-presented identification, thresholded at *p* < 0.001). Next, we implemented the equation i1 + 2*i2 in IMCalc in SPM8 to find all the regions throughout the brain that showed a specific positive mood effect and a self-referential effect. By using the above expression, we were able to find all activated voxels in image 1 (positive vs. neutral mood state), all activated voxels in image 2 (self-generated vs. externally-presented identification), and all voxels which showed activation overlap (i.e., image 3) between the two contrast masks 1 and 2. In this way, we were able to find overlapping as well as adjacent non-overlapping areas within regions that showed both positive mood effects as well as the reality monitoring self-generated identification effects. Using the same procedure, we conducted conjunction analyses between the whole-brain negative mood effect (negative vs. neutral mood state, thresholded at *p* < 0.001) with the whole-brain self-generated identification effect (self-generated vs. externally-presented identification, thresholded at *p* < 0.001) to find regions that showed a specific negative mood self-referential effect.

Finally, we examined brain-behavior correlations within five ROIs, which included our *a priori* mPFC and PCC ROIs; as well as caudate, putamen and parahippocampal regions, for which previous studies have shown both positive mood and source memory effects, as previously mentioned in the Introduction (Adcock et al., 2006; Mitchell and Johnson, 2009; Elward et al., 2015). We, therefore, computed mean beta signal from the functional positive mood activation effect (i.e., the positive vs. neutral mood contrast, thresholded at *p* < 0.001), restricted to each region’s anatomically-defined boundaries. Pearson’s two-tailed correlations were used to examine brain-behavior associations by comparing mean beta signal within the ROIs that showed positive mood sensitive effects with task performance (self-generated identification and externally-presented identification). Outliers were defined as values more/less than 3 SD above/below the mean. Since the mean beta signal was computed from an independent positive mood activation contrast (i.e., the positive vs. neutral mood contrast), and used to interrogate subsequent performance (self-generated and externally-presented accuracy) based on an entirely different reality-monitoring task, the brain-behavior correlations are unbiased and completely independent of each other.

## Results

### Efficacy of Mood Induction

**Figure 2:**
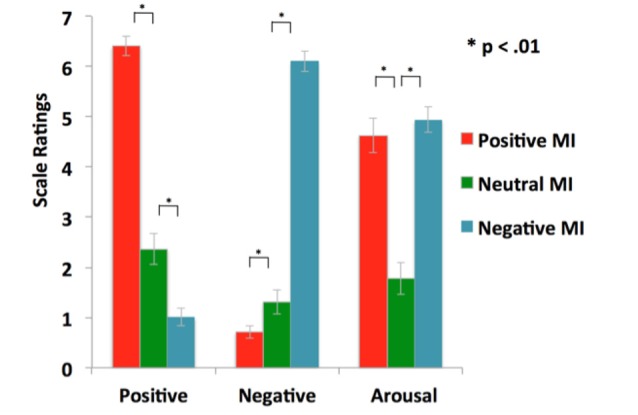
**Mood manipulation check: illustration of successful positive and negative mood inductions (MI) that enhanced the target mood state, relative to the neutral MI**.

Statistical tests confirm the efficacy of our MI protocols. Repeated-measures ANOVAs revealed that participants rated their positive mood higher in the positive MI condition when compared to the neutral MI (*F* = 157.17, *p* < 0.0001), and their negative mood higher in the negative MI when compared to the neutral MI (*F* = 257.73, *p* < 0.0001), confirming that the target mood state was successfully induced (see Table 1, Figure 2). Participants did not differ in their ratings of arousal levels between positive and negative mood states or in ratings of the targeted MI valence magnitude level (i.e., positive rating magnitude for positive MI compared to negative rating magnitude during the negative MI; all *p*’s > 0.10). Together these findings demonstrate the effectiveness of our MI protocols.

**Table 1:** **Mood induction (MI) ratings and reality-monitoring source-memory accuracy**.

	Positive MI	Neutral MI	Negative MI
Positive scale ratings (SD)	6.41 (1.15)	2.36 (1.81)	1.01 (1.02)
Negative scale ratings (SD)	0.71 (0.73)	1.31 (1.40)	6.11 (1.23)
Arousal/activation scale ratings (SD)	4.63 (2.05)	1.78 (1.84)	4.94 (1.50)
% Accuracy self-generated (SD)	83.76 (9.55)	80.53 (10.09)	83.05 (12.26)
% Accuracy externally-presented (SD)	86.51 (13.0)	82.80 (13.45)	83.53 (13.59)
% Total accuracy	85.51 (9.82)	81.67 (13.85)	83.29 (11.05)

### Effects of Mood Induction on Reality Monitoring Accuracy

**Figure 3:**
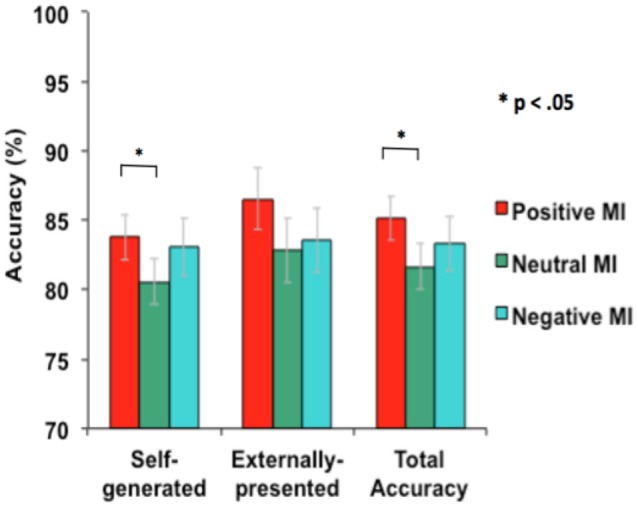
**Mean accuracy during reality monitoring task performance for the three types of MI**.

Mood significantly improved overall source-memory performance. A one-way repeated-measures ANOVA revealed an influence of mood on overall source-memory performance (i.e., accuracy was summated across both self-generated and externally-presented information; *F* = 3.25, *p* = 0.05). Follow-up tests showed that this accuracy difference was driven by participants performing better in the positive mood condition when compared to the neutral mood condition (*F* = 6.55, *p* = 0.01), but not in the negative vs. neutral mood condition (*F* = 0.66, *p* = 0.42). We also found a main effect of mood on reality monitoring self-external accuracy for the positive vs. neutral mood condition (*F* = 6.55, *p* = 0.015), but not for the negative vs. neutral mood condition (*F* = 0.66, *p* = 0.42). Specifically, participants identified significantly more self-generated information (*F* = 5.12, *p* = 0.03) and marginally more externally-presented information (*F* = 3.52, *p* = 0.07) in the positive mood condition when compared to the neutral mood condition (see Figure 3). Together, these findings indicate that a positive mood enhances accurate identification of self-generated information and also marginally enhances correct identification for externally-presented information, contributing to facilitating overall source memory accuracy. By contrast, we did not find any influence of negative mood on either self-generated, externally-presented information or overall accuracy (all *p*’s > 0.20).

### Interaction of Mood Induced Neural Activity With Neural Activity During Reality Monitoring

**Figure 4:**
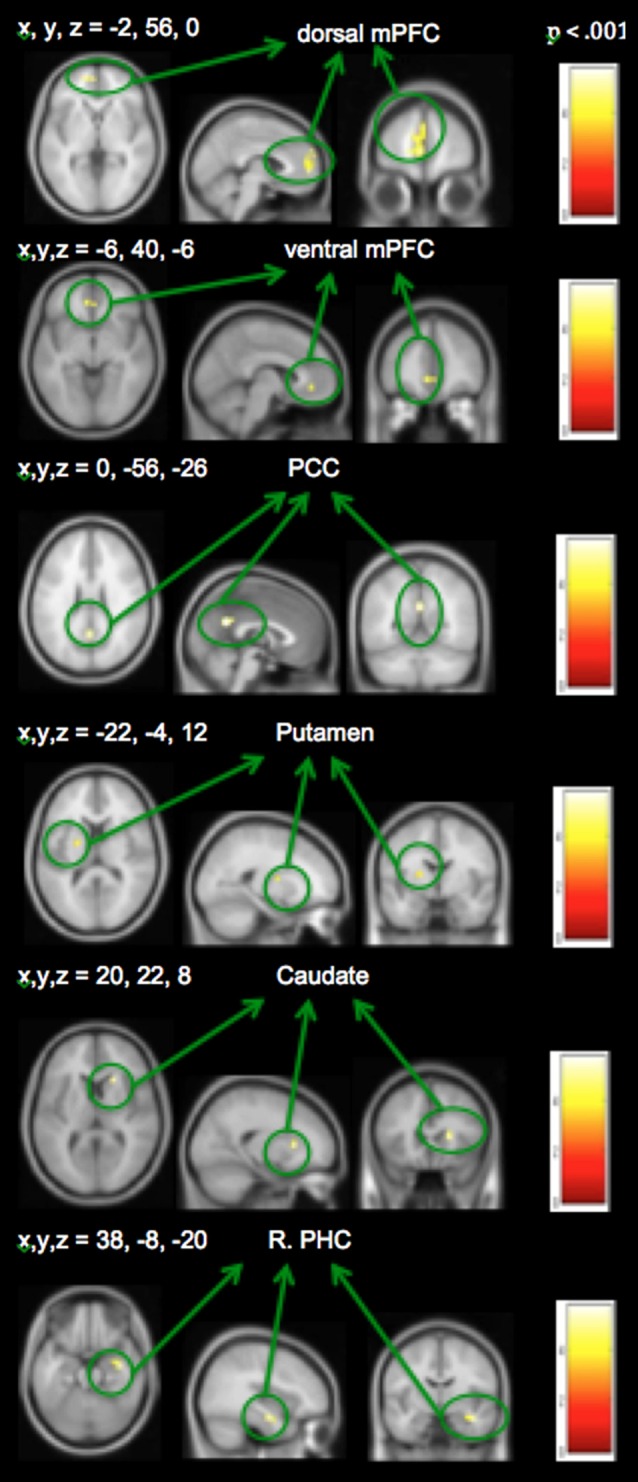
**Positive mood preparatory effect: whole-brain analyses revealing regions showing greater signal during positive vs. neutral mood states**.

Distinct mood states induce neural activity in a variety of brain regions, of which positive mood sensitive regions when compared to a neutral mood, were found in mPFC, PCC and the basal ganglia, among other regions. Our repeated measures ANOVA with MI condition (positive mood, neutral mood, negative mood) as the within-subject factor show all regions revealing positive mood effects, neutral mood effects, and negative mood effects, at a voxelwise *p* < 0.001 threshold, surviving FWE cluster correction at *p* < 0.05, illustrated in Table 2. In order to examine discrete effects of positive mood and negative mood states when compared to neutral mood states, we conducted follow-up ANOVAs that reveal all regions showing greater signal when participants were in positive and negative mood states (see Table 3, Figure 4). Specifically, whole-brain positive mood effects when compared to a neutral mood were found in dorsal and ventral mPFC, PCC, caudate, putamen and parahippocampal cortex (PHC), with the dorsal and ventral mPFC and PCC surviving a FWE cluster corrected extent at *p* < 0.05 (see Table 3, Figure 4). We also found greater signal within left superior temporal gyrus/left PHC (L.STG/LPHC), and basal ganglia (i.e., left caudate and putamen) when participants were in negative mood states compared to neutral mood states (i.e., at our voxelwise *p* < 0.001 threshold, with no clusters surviving the FWE cluster correction).

**Table 2:** **Whole-brain neural activity induced by each mood state**.

Region	BA	Volume (voxels)	Max *Z*	Coordinates		
				*X*	*Y*	*Z*
**A. Main effect of positive mood (*p* < 0.001,**
**with FWE *p* < 0.05 cluster correction, >111 voxels)**
mPFC	10, 9	1347	5.31	−18	50	20
				−8	54	10
R. Lingual gyrus	17, 18	470	5.68	8	−90	−2
L. IFG	47, 38, 22	259	4.55	−52	22	−6
R.IFG	47, 13, 22	202	4.37	56	14	−2
Putamen/Insula	13	191	5.05	36	6	10
				20	10	18
R. SFG	6	183	4.83	2	8	62
				6	6	72
L. Lingual gyrus	17, 18	160	4.10	−4	−98	−8
L. SFG	8	111	4.26	−18	16	46
**Main effect of positive mood (*p* < 0.001, uncorr)**
Parahippocampal gyrus	20	75	4.96	33	−5	−23
**B. Main effect of neutral mood (*p* < 0.001,**
**with FWE *p* < 0.05 cluster correction, >99 voxels)**
R.STG/R.IFG	38, 22, 47	937	5.55	56	14	0
L.MFG	6, 8, 9	582	4.55	−48	18	42
L.IFG/MFG	46, 10	481	4.77	−48	42	6
L.STG	22, 38	290	4.07	−48	12	−4
**C. Main effect of negative mood (*p* < 0.001,**
**with FWE *p* < 0.05 cluster correction, >128 voxels)**
mPFC	10, 32	860	4.97	−10	52	12
SFG	6	128	4.49	−4	12	70
**Main effect of negative mood (*p* < 0.001, uncorr)**
Lingual/Fusiform gyrus	19	79	5.28	30	−76	−20
L. IFG	47, 22	44	4.21	−56	16	−2
Parahippocampal gyrus/Amygdala	28, 35	41	3.98	23	−11	−15

**Table 3:** **Positive vs. Neutral MIs and Negative vs. Neutral MIs**.

Region	BA	Volume (voxels)	Max *Z*	Coordinates		
				*X*	*Y*	*Z*
**A. Positive vs. Neutral mood induction (*p* < 0.001,**
**with FWE *p* < 0.05 cluster correction, >67 voxels)**
Dorsal mPFC	9, 10	246	4.33	−2	56	0
				−8	54	14
Lingual gyrus	19, 18	132	4.37	18	−82	−18
Ventral mPFC	10, 32	99	4.18	−6	40	−6
				2	38	−4
L.IFG/L.STG	22, 47, 38	90	4.13	−52	16	−8
PCC/Precuneus	31	75	3.72	0	−56	26
**Positive vs. Neutral mood induction (*p* < 0.001, uncorrected)**
R. PHC/R.Amygdala	20	63	4.25	38	−8	−20
SFG	6	39	4.14	2	10	64
Caudate	13	28	4.75	20	22	8
L. Putamen	13	27	3.79	−22	−4	12
L. Hippocampus/PHC	35	23	4.08	−28	−14	−14
**B. Negative vs. Neutral mood induction (*p* < 0.001, uncorrected)**
L.STG/L.PHC	35	48	5.08	−42	−30	−8
L. Extra-Nuclear/Caudate	47	47	4.03	−14	30	6

**Figure 5:**
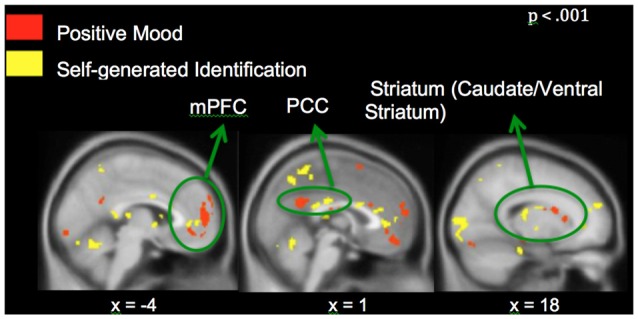
**Whole-brain conjunction analyses illustrating positive mood effect (in red) and self-generated identification effect (in yellow) in medial prefrontal cortex (mPFC), posterior cingulate cortex (PCC) and striatum**.

Neural activation associated with self-generated vs. externally-presented item identification has been reported in several studies of ours and others (Vinogradov et al., 2006, 2008; Schmitz and Johnson, 2007; Murray et al., 2012; Subramaniam et al., 2012b). These regions include dorsal and ventral mPFC and PCC, and we confirm activation of these regions within the current study. Here, we focus on the interactions between recruitment of brain regions during MI and reality monitoring. Therefore, we conducted conjunction analyses to find regions that showed both positive mood effects (positive vs. neutral mood state) and self-generated accuracy effects (self-generated vs. externally-presented item identification). We found three regions within mPFC, PCC and striatum that were sensitive to both positive mood effects (red voxels) and self-generated item identification effects (yellow voxels; see Figure 5). We did not find any regions that demonstrated both negative mood effects (negative vs. neutral mood state) and self-generated item identification effects (self-generated vs. externally-presented item identification).

**Figure 6:**
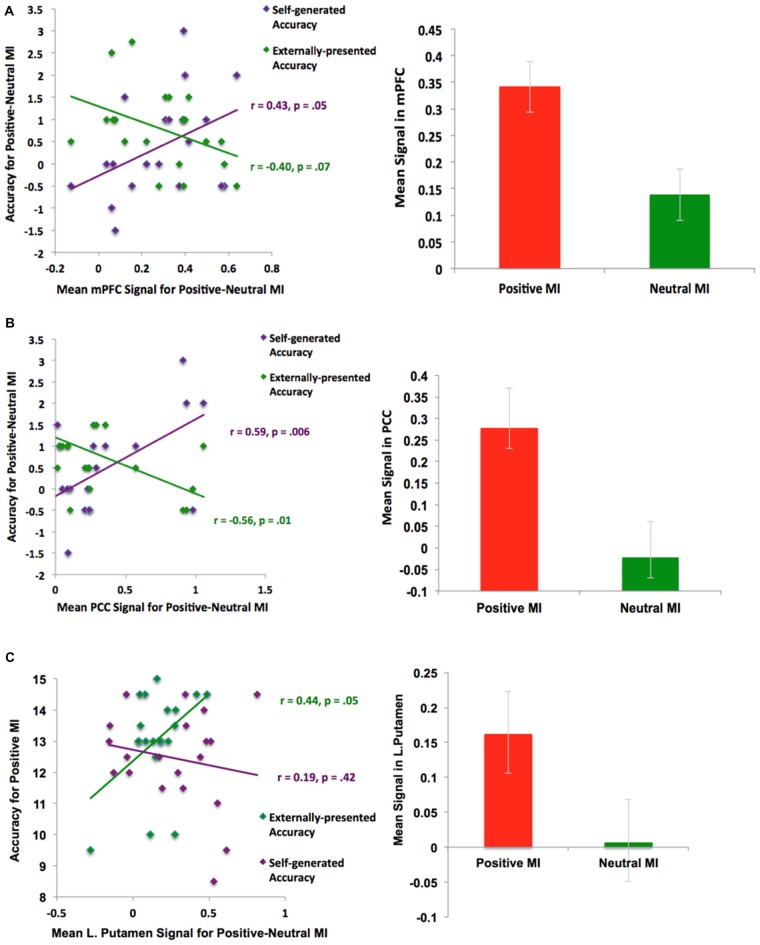
**ROI Analyses: Positive mood sensitive regions predict better reality monitoring task performance within (A)** mPFC, **(B)** PCC and **(C)** basal ganglia/putamen.

In support of our *a priori* hypothesis, subsequent ROI analyses revealed that signals within the mPFC and PCC during the positive vs. neutral MI correlated with better subsequent self-generated item identification (see Figure 6). Interestingly, we also found that signal within the left putamen correlated with better identification of externally-presented information. We did not find any outliers for any correlations. We also did not find any significant correlations between age with behavior or neural signal in the present study (all *p*’s > 0.15). Together, these findings indicate that distinct neural networks seem to support “self” and “external” judgments during the positive MI; while activation within mPFC and PCC during the positive MI predicted better accuracy for self-generated information (but not externally-presented information), activation within the putamen correlated with externally-presented item accuracy (but not self-generated item accuracy).

## Discussion

### Positive Mood Enhances Reality Monitoring Accuracy

This is a first-in-kind study in which we demonstrate the neural plasticity of positive mood states on improving critical cognitive reality-monitoring functions during identification of previously self-generated and externally-presented information. Relative to a neutral mood, when participants were in a positive mood, they correctly identified significantly more self-generated information and marginally more externally-presented items. By contrast, the negative mood condition had no effect on reality monitoring task performance. Hence, the principal focus of this article is on the impact of positive mood on reality monitoring.

### Neural Mechanisms of Positive Mood-Cognition Enhancements During Reality Monitoring

Our conjunction analyses confirmed that mPFC, PCC and caudate/ventral striatum, showed sensitivity to both positive MI (red voxels) and identification of self-generated items (yellow voxels) during the reality monitoring task (Figure 5). We did not find voxel activation overlap within the three regions, suggesting that specific areas within mPFC were particularly sensitive to positive mood effects and other subdivisions within mPFC were more sensitive to self-generated effects, yielding adjacent non-overlapping voxels within these regions. Consistent with our behavioral findings, our fMRI results did not yield any effect of negative mood states on reality monitoring. We found a main effect of a negative mood within mPFC and parahippocampal gyrus/amygdala (Table 2); however, we did not find any interaction between negative mood states (when compared to a neutral mood) and self-generated item identification from our conjunction analyses.

### Brain-Behavior Correlations During the Positive Mood Induction

When people were in a positive relative to a neutral mood state, they showed activation in several regions that correlated with better subsequent reality monitoring item identification. First, we found that activation within the mPFC and PCC facilitated better subsequent identification of self-generated information (Figure 6). This set of findings is consistent with past research showing that positive mood states and positive rewarding stimuli enhance preparatory activity within the mPFC to facilitate subsequent cognitive performance (Knutson and Cooper, 2005; Subramaniam et al., 2009, 2012a), and that the mPFC supports reality monitoring task performance (better identification of self-generated items; Subramaniam et al., 2012b). In our previous fMRI study of problem-solving (Subramaniam et al., 2009), the PCC also showed increased activity during a preparation period preceding problem-solving, and was a positive mood-sensitive region that facilitated subsequent problem-solving. Second, we found that activation within the basal ganglia/striatum (i.e., left putamen) facilitated better identification of externally-presented information. The basal ganglia/striatum with its high density of dopamine receptors is also known to be central to both the identification and maintenance of memories (McNab and Klingberg, 2008), and to positive stimuli (Knutson and Cooper, 2005).

In summary, we have strongly demonstrated that positive mood is reliably associated with preparatory states that increase signal within mPFC and PCC, and to a lesser extent within the striatum, and that this modulation leads to better reality monitoring. We are not arguing that mPFC, PCC and striatal activation represent neural correlates of positive mood, or that positive mood states induce reality monitoring. We conclude that a positive mood is one factor that enhances activity in mPFC, PCC and striatum, which then mediates the shift towards better reality monitoring. Reality-monitoring requires working memory and cognitive control processes, which are multifaceted processes, involving the recruitment of frontal regions—including ACC/mPFC as well as bilateral prefrontal cortices, implicated in controlling attention encoding of relevant information from environmental stimuli into working memory processes; and switching attention to select the correct response (Kondo et al., 2004; Hedden and Gabrieli, 2006). The specific mechanism by which a positive mood facilitates reality monitoring abilities is not known. Previous research indicates that the mPFC/ACC is implicated in self-referential processes (Cabeza et al., 2004) as well as in general attention and cognitive control processes, involving controlling and switching attention to select the correct response (Kondo et al., 2004; Hedden and Gabrieli, 2006) as well as being modulated during memory and reward decision-making (O’Doherty, 2011; Euston et al., 2012). There is also an abundance of evidence indicating that people in a positive mood state are better able to modulate attention (Gasper and Clore, 2002; Rowe et al., 2007), working memory (Ashby et al., 1999, 2002) and cognitive control processes (Dreisbach and Goschke, 2004). According to Ashby et al. (1999) neuropsychological model, many of these cognitive-enhancing effects of a positive mood are due to increased dopamine release in the PFC and striatum. Further evidence from physiologic and computational neuroscience research additionally indicate that dopamine release (e.g., during positive mood states) can facilitate long-term potentiation, making memories more robust if it is already available at the synapse when neurons fire (Otmakhova and Lisman, 1996; Gruber et al., 2006). These data suggest that regions which are implicated in mnemonic encoding and retrieval processes—specifically in source-memory retrieval such as the parahippocampal cortices, (Mitchell and Johnson, 2009; Elward et al., 2015) regions which have dopaminergic innervations—such as the striatum—which are activated during positive mood states may also help to predispose and facilitate overall memory recognition processes (Adcock et al., 2006; Mitchell and Johnson, 2009; Elward et al., 2015). Specifically, dopamine release during positive mood states is thought to enhance the initiation and maintenance of mnemonic processes by protecting information from noise or distraction via enhancement of prefrontal-striatal/basal ganglia interactions (Gruber et al., 2006; McNab and Klingberg, 2008). Thus, it is possible that a positive mood may enhance reality monitoring abilities either by enhancing attention and long term potentiation of relevant information from environmental stimuli into working memory processes; and/or enhancing the switching of attention to the reality monitoring task to enable selection of the correct response.

### Caveats and Considerations

In our fMRI study, the MI reality monitoring task relies on each participant’s ability to imagine positive, neutral and negative past experiences through autobiographical recall. The positive and negative MI were both successful at increasing positive and negative mood states respectively, in relation to the neutral mood condition. While the negative MI did not impede reality monitoring when compared to a neutral mood, our behavioral and fMRI findings indicate that a negative mood did not enhance reality monitoring processes either.

The strength of this paradigm is that despite different MI stimuli (i.e., each participant recalled his/her own subjective experiences to elicit positive, neutral and negative mood states), mPFC and PCC regions were consistently activated, surviving whole-brain FWE cluster corrections, to reveal positive mood effects across all participants, relative to the neutral MI condition. Both positive and negative mood states also increased participants’ arousal ratings, relative to the neutral mood condition, and positive and negative mood states did not differ in magnitude or arousal levels. Therefore, the positive mood effects we observed in mPFC and PCC cannot simply be the result of enhanced valence magnitude or arousal levels; if this were the case, then we would also expect to observe activation within these regions during the negative mood condition relative to the neutral mood condition, which we did not find.

It is well-established that mPFC and PCC are also active during the default state (Raichle et al., 2001). It does not appear that the positive mood associated changes in mPFC and PCC that we observed in the present study reflect modulation of the default state network, as this would imply that these same regions would de-activate during task-directed behaviors, which was not the case (i.e., mPFC and PCC both revealed increased activation from baseline during identification of self-referential information).

Additionally, it must be noted that the location of the dorsal mPFC that shows positive mood sensitive effects is in a very different location from subgenual cingulate regions that mediate depression and anxiety (Mayberg et al., 1999; Ochsner et al., 2002; Kohn et al., 2014). Prior studies have reported on the reciprocal nature of subgenual cingulate and dorsal ACC/mPFC activation; specifically activation within the subgenual cingulate is increased during depression, while increased activation within the dorsal mPFC/ACC (the positive mood sensitive region shown in our study) is found when people remit from depression and sadness (Mayberg et al., 1999; Ochsner et al., 2002). On this note, the mid-cingulate cortex, which has shown to be sensitive to conflict and error detection (Carter et al., 1998; Botvinick et al., 2004; Kerns et al., 2004), is another region that is in a very different location from the dorsal mPFC region we report here which shows sensitivity to positive mood and reality monitoring self-generated recognition effects.

It is also possible that a positive mood may enhance the willingness to judge information as “self-generated” as opposed to “externally-presented;” however in this case, we would find that a positive mood would then decrease the accurate identification for externally-presented information as a result of external judgments being more likely to be misattributed as “self”. Rather, our behavioral data indicate that a positive mood marginally enhances identification of external information, thus contributing to enhanced overall reality monitoring accuracy. This raises a final possibility as to whether the reality monitoring enhancements we observed are the result of a positive mood or are instead driven by a self-referential memory effect resulting from general autobiographical recall processes during the MI. Here again, if this were the case, then we would expect to observe these enhancements during the negative mood condition, which we did not find.

### Conclusions and Future Directions

In conclusion, we found that when people are in a positive mood, they show increased activity within the mPFC, PCC and striatum which precedes and potentiates subsequent reality monitoring task performance. Activity within mPFC and PCC predicted better identification of self-generated information while activity within the putamen predicted better subsequent externally-presented item identification. By contrast, a negative mood did not impact reality monitoring abilities. Therefore, future studies are needed to investigate the specific cognitive processes involved in prefrontal-cingulate-striatal interactions that mediate the shift towards better self-generated processing or towards better externally-presented item identification when people are in a positive mood state. These findings may also open new paths for the development of novel biological-cognitive treatments for patients suffering from psychiatric illness. Individuals with schizophrenia, for example, show reality monitoring impairments associated with mPFC hypoactivation, as well as a range of cognitive deficits (Fisher et al., 2008; Vinogradov et al., 2008; Subramaniam et al., 2012b). Intensive computerized cognitive training has been shown to enhance activity in the mPFC in a manner associated with better reality monitoring (Subramaniam et al., 2012b). If positive MI techniques can also show similar enhancing effects in schizophrenia patients, it might suggest that behavioral treatments which increase hedonic capacity and/or harness hedonic mechanisms in the brain may help to generate improved cognitive performance via enhancement of signal within mPFC, PCC and the striatum.

## Author Contributions

KS is responsible for all the collection, analyses and interpretation of the behavioral and neuroimaging data and wrote the manuscript; JG helped with collection and analyses of the fMRI data; PS and AS helped with analyses of the behavioral data; DHM provided advice and consultation on the design and analyses of the fMRI data; SN edited the manuscript and provided advice on the design and analyses of the fMRI data; SV edited the manuscript.

## Conflict of Interest Statement

The authors declare that the research was conducted in the absence of any commercial or financial relationships that could be construed as a potential conflict of interest.

## Footnotes

^1^http://www.fil.ion.ucl.ac.uk/spm/software/spm8/

^2^http://arxiv.org/abs/1606.08199
