# Supplementary figures and images for: Neural Mechanisms of Positive Mood Induced Modulation of Reality Monitoring
Source: Front Hum Neurosci. 2016 Nov 15;10:581. doi: 10.3389/fnhum.2016.00581 (PMC5108806; doi:10.3389/fnhum.2016.00581)

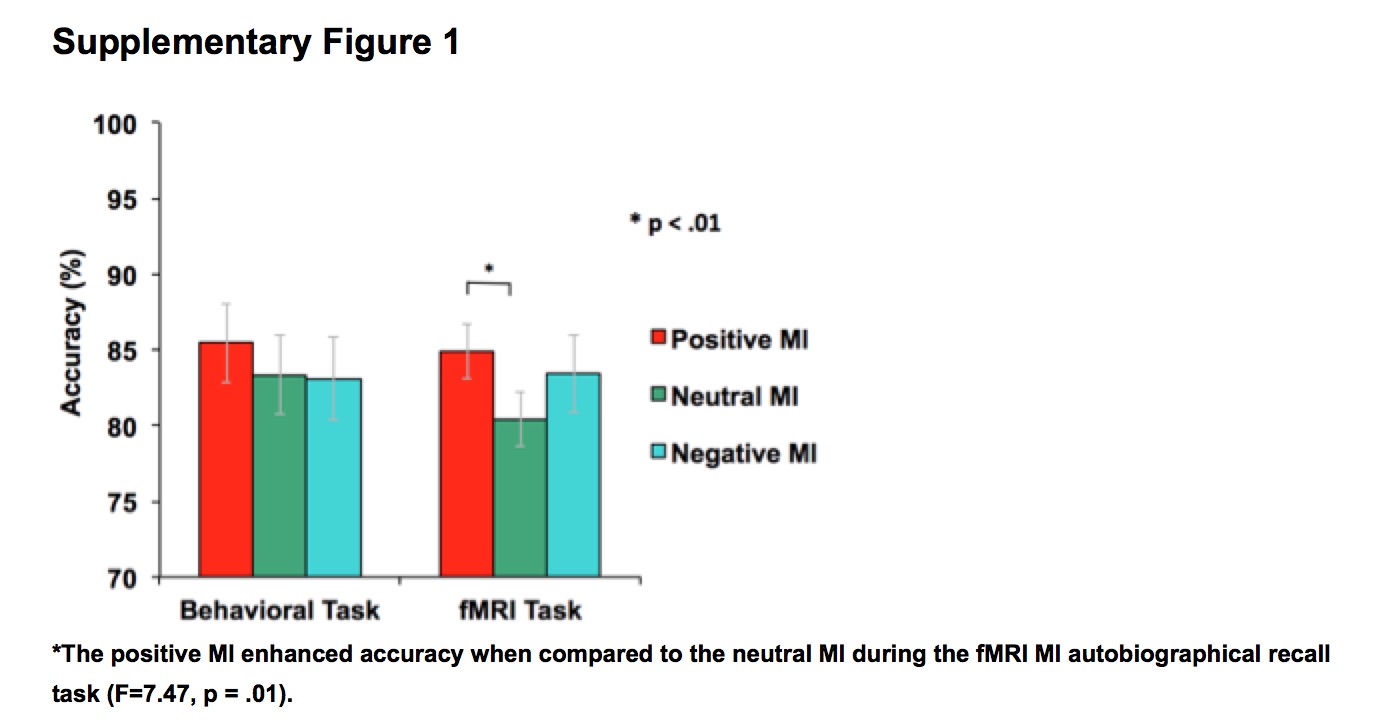

Supplement: Supplementary file 1 [file Image_1.jpeg]
