# Supplementary material for: Neural Mechanisms of Positive Mood Induced Modulation of Reality Monitoring
Source: Front Hum Neurosci. 2016 Nov 15;10:581. doi: 10.3389/fnhum.2016.00581 (PMC5108806; doi:10.3389/fnhum.2016.00581)
Supplement: Supplementary file 2 [file Table_1.docx]

| **Behavioral Mood Induction Reality Monitoring Task: MI Ratings after IAPS Pictures** | | | |
| --- | --- | --- | --- |
|  | **Positive MI** | **Neutral MI** | **Negative MI** |
| **Positive Scale Ratings** | 5.72 | 3.19 | 1.52 |
| **Negative Scale Ratings** | 0.67 | 1.78 | 5.46 |
| **Arousal/Activation Scale Ratings** | 3.69 | 2.71 | 4.43 |
| **fMRI Mood Induction Reality Monitoring Task: MI Ratings after Autobiographical Recall** | | | |
| **Positive Scale Ratings** | 6.96* | 1.70 | 0.60 |
| **Negative Scale Ratings** | 0.74 | 0.93 | 6.62* |
| **Arousal/Activation Scale Ratings** | 5.38 | 1.03 | 5.35 |

**Supplementary Table 1: Mood Inductions Ratings**

**^*We found that using personal autobiographical recall was a better mood induction technique at enhancing positive mood and negative mood states when compared to using pictures from the International Affective Pictures System (IAPS) IAPS (*all p’s < .01).^**
